# Whole-Brain Dimensions of Intrinsic Connectivity Capture Modality-Specific and Heteromodal Language Representations

**DOI:** 10.1523/JNEUROSCI.1876-24.2025

**Published:** 2025-04-30

**Authors:** Lidon Marin-Marin, Susanne Eisenhauer, Tirso R. J. Gonzalez Alam, Daniel S. Margulies, Jonathan Smallwood, Elizabeth Jefferies

**Affiliations:** ^1^Department of Psychology, University of York, York YO10 5NA, United Kingdom; ^2^York Neuroimaging Centre, Innovation Way, York YO10 5NY, United Kingdom; ^3^Basque Centre of Cognition, Brain and Language (BCBL), Donostia, Basque Country 20009, Spain; ^4^School of Psychology, Bangor University, Bangor LL57 2AS, United Kingdom; ^5^Centre National de la Recherche Scientifique (CNRS), Université de Paris, Paris 75016, France; ^6^Department of Psychology, Queen's University, Kingston, Ontario K7L 3N6, Canada

**Keywords:** auditory, connectivity, connectivity dimensions, language comprehension, visual, whole-brain activity

## Abstract

Comprehension of spoken and written language involves a hierarchical sequence of modality-specific and heteromodal processes. While these have been localized to different regions, modality-selective responses extend beyond them, implicating large-scale network organization in language comprehension. Dimensions of whole-brain connectivity, derived from intrinsic activity, have been proposed as a general organizing framework for cognition. Here, we test their utility in accounting for the spatial distribution of task-evoked activity during language comprehension. We investigated brain activity in human males and females in response to psycholinguistic variables linked to input processing and meaning in a sentence comprehension task presented both visually and auditorily. Macroscale patterns of brain activity were similar across modalities for sentence-level and semantic variables, but effects of orthographic and phonological distance were negatively correlated between modalities. The first dimension, separating heteromodal and unimodal cortices, showed no differences across modalities for sentence processing and semantic variables and opposite effects of word length and orthographic/phonological distance for spoken and written words, supporting the notion that higher-order processing requires heteromodal resources different to those linked to input processing. The second dimension, separating auditory–motor and visual processes, showed an asymmetry in the recruitment of the unimodal systems—listening to long and semantically dissimilar words involved stronger recruitment of primary auditory–motor regions and low visual engagement. These findings show that the language system is organized according to large-scale axes of intrinsic connectivity, with psycholinguistic processes varying systematically along whole-brain dimensions. This supports the view that language comprehension reflects general principles of cortical organization.

## Significance Statement

Whole-brain dimensions of functional connectivity, derived from intrinsic activity, have been proposed as a general organizing framework for cognition, yet their relevance to specific, ecologically meaningful tasks remains underexplored. Here, we show that these macroscale patterns account for key differences and commonalities in how the brain processes spoken and written language. Our findings reveal a principled division between modality-general semantic and sentence-level processes versus modality-specific input effects, structured along the first two axes of intrinsic connectivity. These results demonstrate that the language system aligns with domain-general principles of cortical organization.

## Introduction

Comprehension of spoken and written language entails a series of hierarchical yet interactive processing steps, starting with acoustic and visual processing of the input in primary sensory regions, followed by phonetic and orthographic processing and then syntactic and semantic decoding of words, phrases, and passages in heteromodal regions such as the anterior temporal lobe (ATL; Price, 2012). This shows that our ability to extract meaning from spoken and written stimuli emerges from both unimodal processes, which are specific to one input modality such as sound or vision, and heteromodal processes, which are independent of input modality (Uddén et al., 2022). However, this focus on the function of individual brain regions does not emphasize systematic change in function linked to their anatomical location (Margulies et al., 2016) or the way that cognition may be influenced by whole-brain states, with different functions being supported by the same regions across contexts (Wang et al., 2024).

Many models of language processing assume that input modality primarily affects unimodal processing stages, while semantic and syntactic processes are heteromodal (Friederici, 2011; Price, 2012; Carreiras et al., 2014; Deniz et al., 2019). Yet modality-selective responses are seen at the macroscale (Regev et al., 2013), potentially reflecting differences across spoken and written language inputs in processing demands (Chen et al., 2023). Auditory stimuli are dynamic and transient in nature—and these fundamental differences between auditory and visual inputs are likely to influence the topographical landscape of brain activity patterns underpinning language in ways not fully captured by the unimodal–heteromodal distinction (Cohen et al., 2009; Robinson et al., 2018).

**Figure 1.**
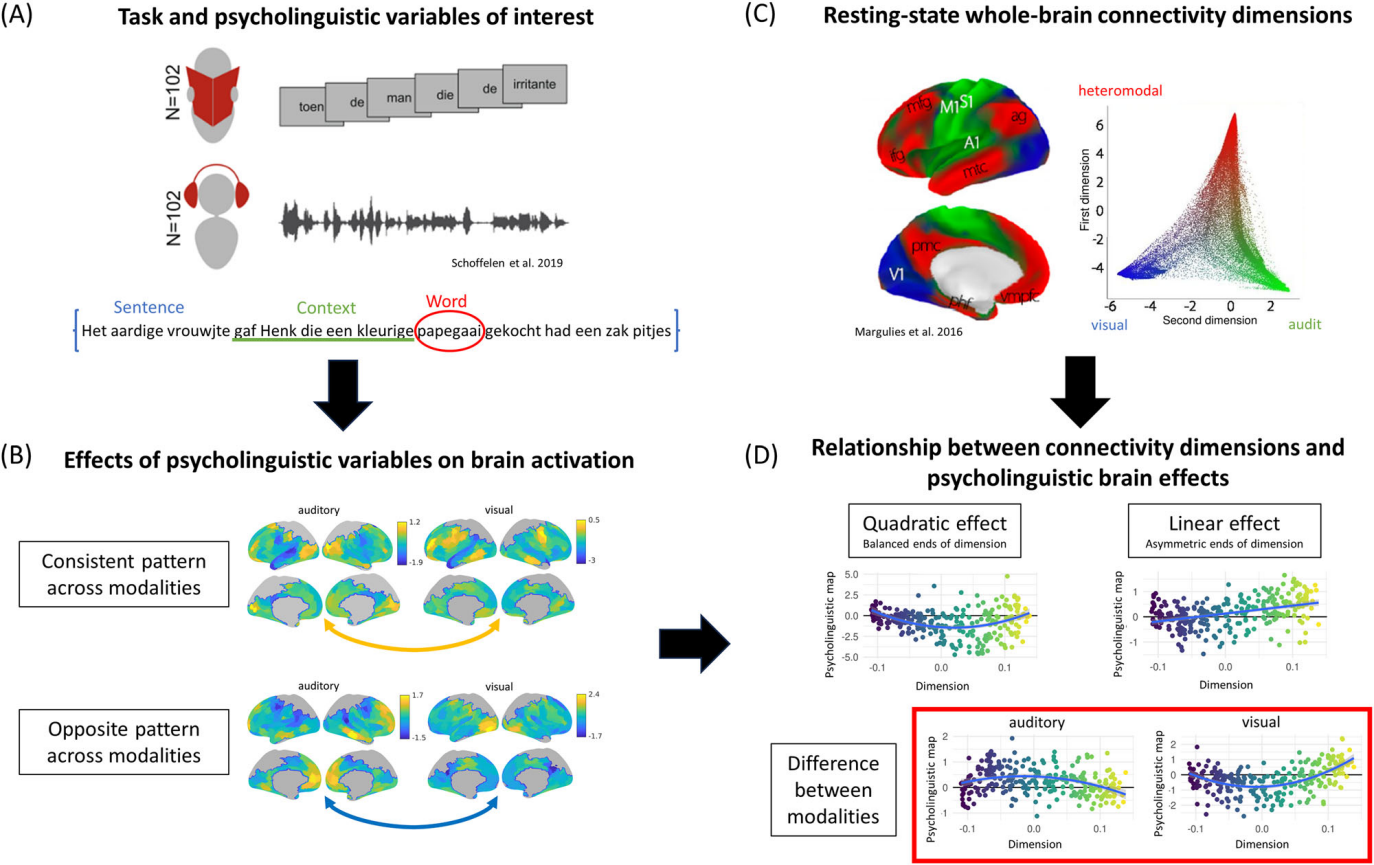
Schematic overview of the procedure. ***A***, Participants were instructed to focus on understanding written and spoken sentences presented word by word. We examined variables representing different linguistic levels of language processing: sentence, context, and word level. ***B***, Cortical maps represent the strength and direction of the effects of those variables on brain activity for each modality separately, averaged for 400 brain parcels (parcels missing due to field of view); these maps were compared across modalities using correlations (results in Similarity between modalities in the effects of psycholinguistic variables on brain activation). ***C***, The first two dimensions of intrinsic connectivity that explain the most variance in functional organization. Each dot represents the dimensional value of each brain node in the two dimensions, obtained from similarity of connectivity profiles among node pairs using the procedure explained in Materials and Methods, Modality differences in the relationship between variables and connectivity dimensions. The first dimension represents the gradual change from regions associated with unimodal to heteromodal processing, with intermediate values related to attention, salience, and cognitive control, while the second dimension separates visual from auditory and somatomotor systems (more details in Materials and Methods, Modality differences in the relationship between variables and connectivity dimensions). ***D***, The effect of these dimensions on activation associated with each psycholinguistic variable was investigated using linear and quadratic models for each modality separately (Intramodality relationship with the first connectivity dimension capturing the heteromodal–unimodal distinction and Intramodality relationship with the second connectivity dimension capturing the distinction between visual and auditory–motor cortices). Differences between modalities were modeled as an interaction term in models including both visual and auditory data (Modality differences in the relationship between psycholinguistic variables and connectivity dimensions).

Although some research has focused on localizing brain areas implicated in language, e.g., lateral temporal and prefrontal regions, comprehension can also be described in terms of responses within large-scale networks (Hagoort and Indefrey, 2014) and whole-brain states (Wu et al., 2022; Aliko et al., 2023). Recent work suggests that these brain states are captured by the principal dimensions of brain connectivity, often referred to as “gradients” (Margulies et al., 2016; Fig. 1*C*): these are extracted by dividing the brain into parcels, computing the time-series correlations between every pair of parcels, and decomposing this connectivity matrix to identify dimensions that correspond to key functional distinctions. The first dimension of intrinsic connectivity captures the transition from sensory–motor systems, through attention networks and frontoparietal control systems to the default mode network (DMN); it therefore has unimodal systems at one end and heteromodal regions at the other. A recent study found that activation related to visual, orthographic, and lexical properties gradually increased toward the sensory end of this dimension across the whole brain (Eisenhauer et al., 2024). Greater separation between unimodal and heteromodal cortices on this first dimension is also related to the retrieval of stronger semantic associations (Wang et al., 2020; Gao et al., 2022), effects of coherence in naturalistic speech (Morales et al., 2022; Wu et al., 2022), and individual differences in semantic performance (Gonzalez Alam et al., 2022; Shao et al., 2022). The second dimension of intrinsic connectivity captures the separation of the auditory and somatomotor cortex from visual regions and predicts performance on picture semantic judgments (Shao et al., 2022); this dimension is expected to be related to unimodal aspects of language.

We investigated how these dimensions of intrinsic connectivity capture the organization of activation during reading and listening, exploring word-, context-, and sentence-level effects in a large sample (Schoffelen et al., 2019). While context- and sentence-level effects are expected to be consistent across modalities (Uddén et al., 2022), the two connectivity dimensions might relate differently to word-level effects. A linear relationship between a connectivity dimension and activation linked to a psycholinguistic variable is expected to delineate gradual functional change and opposing effects of the variable in brain regions falling at the ends of the dimension (Fig. 1*D*, “Linear effect”). In contrast, quadratic effects indicate nonlinear variation, such that regions at both ends of the connectivity dimension show similar functional responses, while the strongest or weakest responses are found in intermediate regions suggesting more rapid functional transitions (Fig. 1*D*, “Quadratic effect”). We interpret these patterns as reflecting either processing asymmetries—where activation shifts from one end of the dimension to the other—or balanced engagement of both ends of a dimension, when heteromodal and unimodal or visual and auditory–motor systems are jointly recruited. In this way, we situate language hierarchies and modality differences within a whole-brain state space characterized by dimensions of intrinsic connectivity, testing the view that language organization at the macroscale aligns with holistic trends in cortical organization.

## Materials and Methods

### Data and participants

We used publicly available task-fMRI data of 204 right-handed Dutch participants (100 males, mean age of 22 years, range of 18–33) from the MOUS (“Mother Of Unification Studies”) dataset (Schoffelen et al., 2019). During the fMRI task, 102 participants read and the other 102 listened to sentences in Dutch. Participants also read or listened to noncoherent word lists created by scrambling the words from the sentences. Out of a total of 360 sentences and their corresponding word lists, each participant was presented with 60 sentences and 60 noncorresponding word lists during the fMRI task, and 20% of them were immediately followed by a “yes”/“no” question about their content (e.g., “Did grandma give a cookie to the girl?”). All sentences and word lists consisted of 9–15 words, and the presentation rate of the visual stimuli was determined in relation to the duration of the spoken sentences and word lists (audiodur), also considering the number of letters and words of the whole sentence (sumnletters; nwords), and letters within each word (nletters), using the following formula: (nletters/sumnletters) × (audiodur + 2,000 − 150 × nwords). We excluded a total of 10 participants from the analyses (three of the visual version of the task and seven of the auditory) because their task-fMRI images were accidentally obtained with a different phase encoding direction. The original project was approved by the local ethics committee (CMO – the local “Committee on Research Involving Human Subjects” in the Arnhem–Nijmegen region) and followed the guidelines of the Declaration of Helsinki (Schoffelen et al., 2019).

### MRI preprocessing

MRI data acquisition protocol and parameters can be found in the original article describing the MOUS dataset (Schoffelen et al., 2019). Task-fMRI data were preprocessed and analyzed following the procedure used in a previous study (Eisenhauer et al., 2024), accessible in Open Science Framework (https://osf.io/5fxbd/), using FMRIB’s Software Library (FSL; version 6.0). Images were corrected for motion using MCFLIRT (Jenkinson et al., 2002), the slice-timing–corrected brain tissue was extracted, a spatial smoothing with a 6 mm full-width-half-maximum Gaussian kernel and high-pass filtering at 100 s was applied, and FLIRT was used for linear registration (Jenkinson and Smith, 2001; Jenkinson et al., 2002). Due to limited field of view for the task-fMRI images that could reduce the accuracy of registration, data were first registered with six degrees of freedom to a brain-extracted slice of each subject’s resting-state fMRI, which had full field of view. Then, task-fMRI images were registered to the brain-extracted T1–weighted anatomical brain images using linear boundary-based registration, which were registered to the MNI152 standard space with 12 degrees of freedom.

### General approach

**Figure 2.**
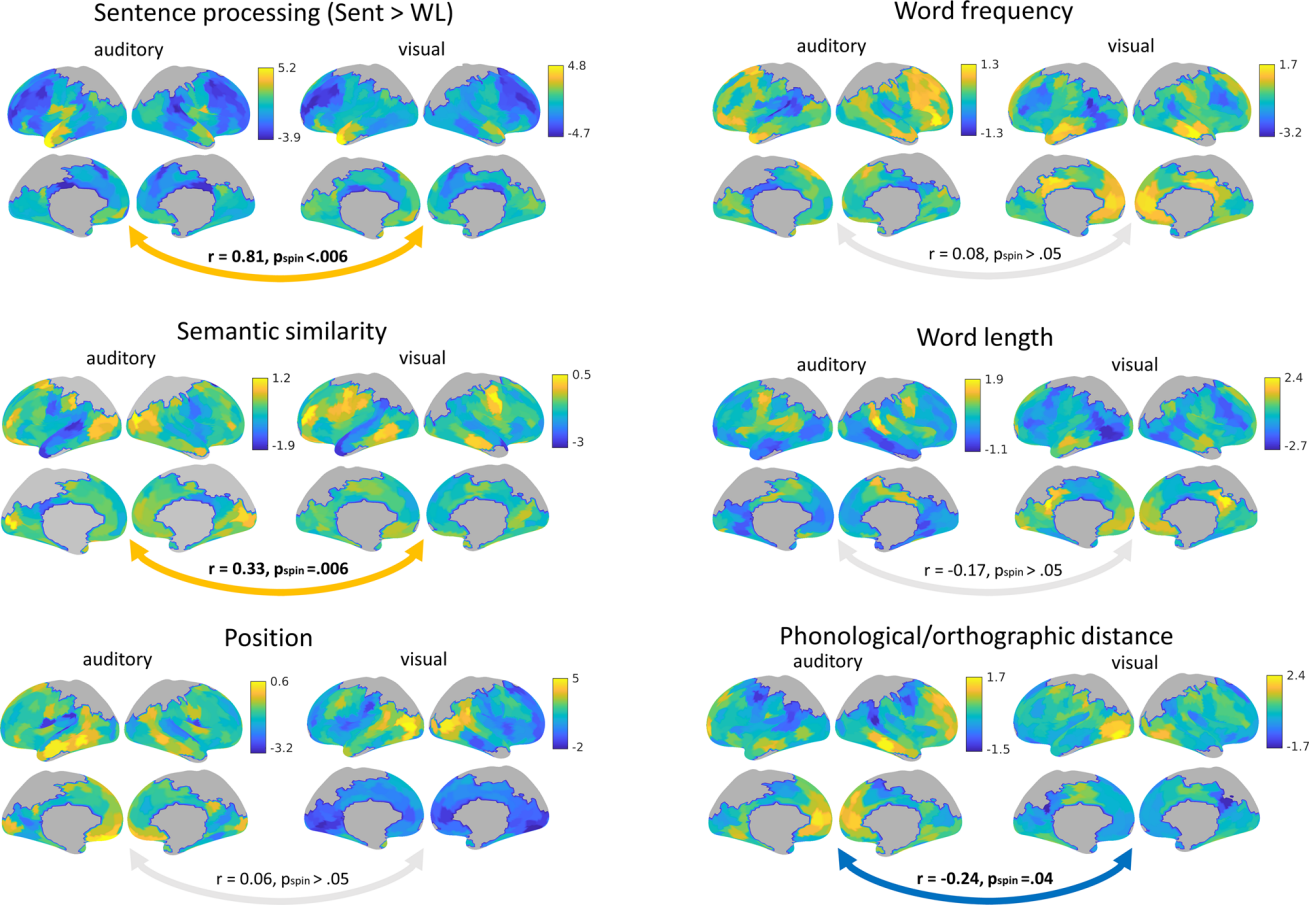
Maps of the macroscale effects of psycholinguistic variables on brain activation. Significant correlations between modalities are indicated in bold (spin permutation, FDR corrected), with a blue arrow for negative and orange for positive. Reduced field of view resulted in lack of data in brain regions colored in gray. Sent, sentences; WL, word lists.

**Figure 3.**
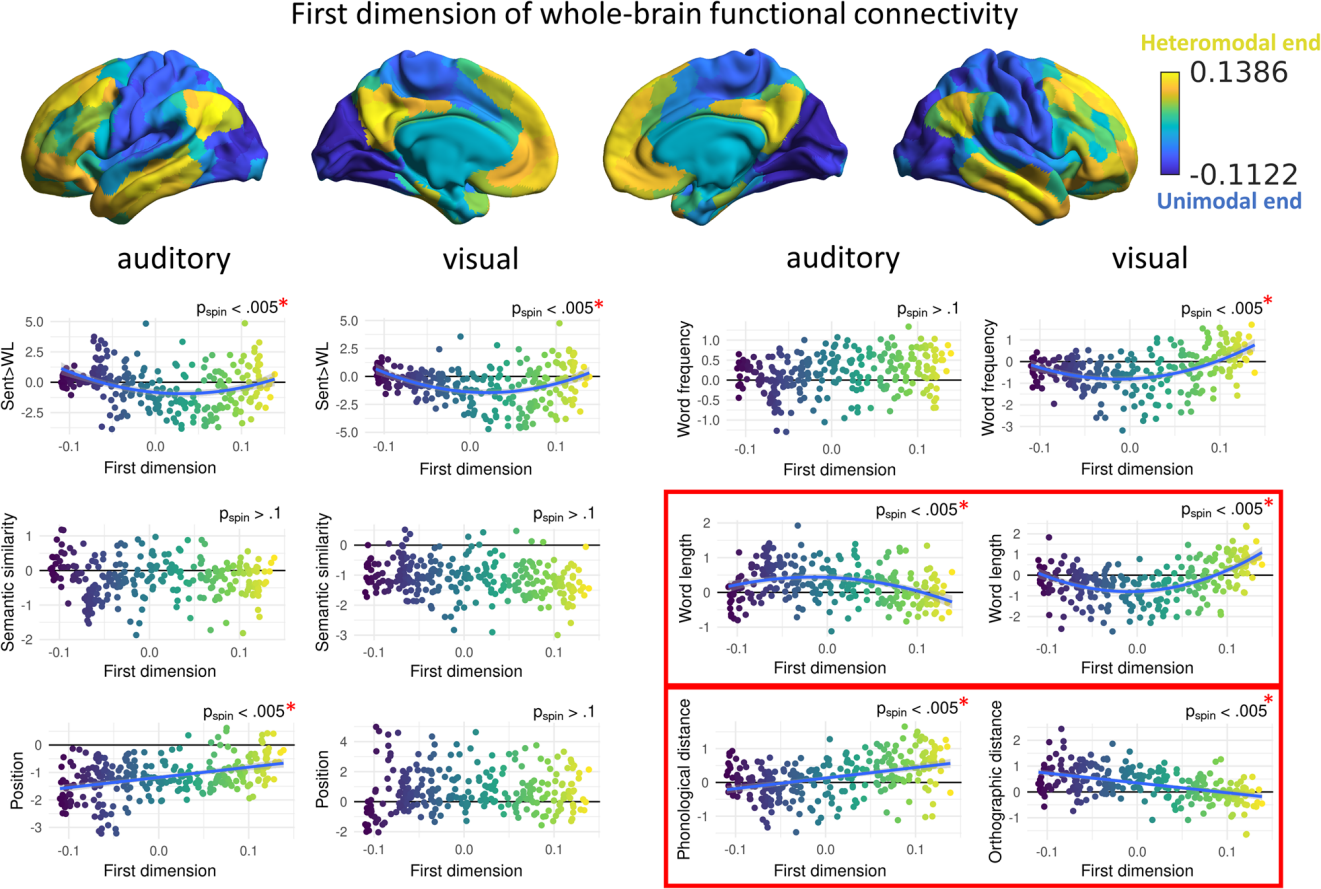
Dependence of psycholinguistic brain effects on the first dimension of whole-brain connectivity. Dots represent parcels in the brain. Red asterisks indicate significant relationships intramodality; red squares indicate significant intermodality differences. Quadratic models are used for representation if the comparison between the linear and quadratic model for that variable and the main effect of the quadratic term were statistically significant (i.e., the nonlinear model explained better the relationship); otherwise, linear models are represented.

**Figure 4.**
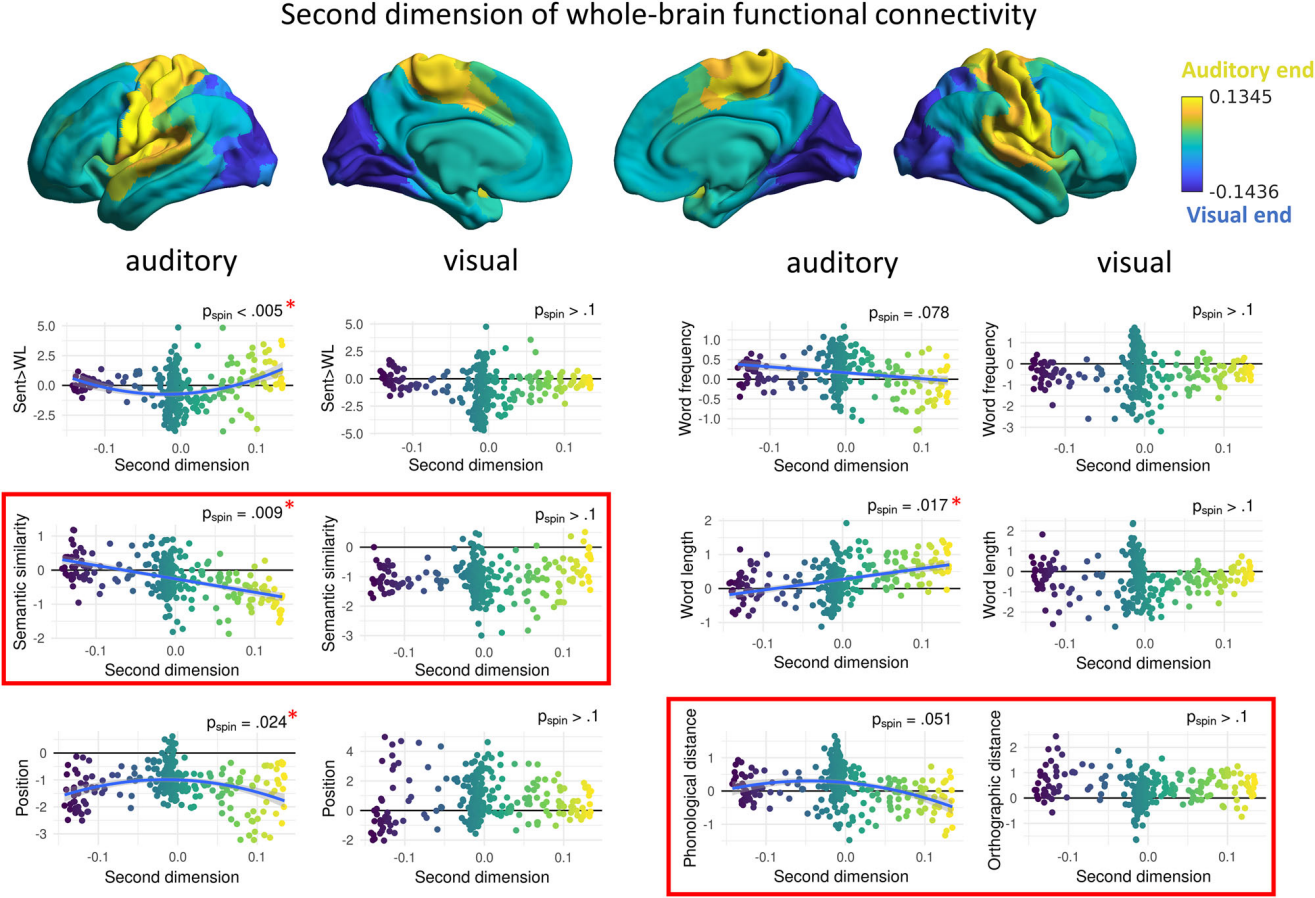
Dependence of psycholinguistic brain effects on the second dimension of whole-brain connectivity. Dots represent parcels in the brain. Red asterisks indicate significant relationships intramodality; red squares indicate significant intermodality differences. Quadratic models are used for representation if the comparison between the linear and quadratic model for that variable and the main effect of the quadratic term were statistically significant (i.e., the nonlinear model explained better the relationship); otherwise, linear models are represented.

Our main aim was to examine whether the two principal dimensions of whole-brain connectivity involved in language processing—one distinguishing heteromodal from unimodal regions (Dimension 1) and the other separating auditory–motor from the visual cortex (Dimension 2)—show different patterns of response across a range of psycholinguistic variables and depending on the sensory modality (auditory vs visual). We first used general linear models (GLMs) to estimate the effects of the variables representing different linguistic processing levels on brain activation (sentence, context, and word level; Fig. 1*A*) at the subject level and then carried out higher-level group mean analyses. As a result, we obtained macroscale brain cortical maps representing the strength and direction of psycholinguistic effects for each modality separately (Figs. 1*B*, 2). To assess whether each variable had similar effects on brain activity across modalities, we correlated the auditory and visual group-level maps across parcels (Figs. 1*B*, 2), generating permuted maps that had preserved spatial autocorrelation to assess statistical significance (spin–permutation) and correcting for multiple comparisons. Next, we examined how strongly each psycholinguistic variable’s effect map was linked to the first two dimensions of whole-brain connectivity (Margulies et al., 2016; Fig. 1*C*). We explored both linear and quadratic models, which allowed us to distinguish when the ends of the dimensions showed opposing or similar responses, indicating gradual functional change and asymmetry between the ends of the dimension or rapid functional change and balance between the ends, respectively. We used the same spin–permutation procedure to assess statistical significance (Figs. 1*D*, 3, 4). Finally, to investigate if these associations between connectivity dimensions and the neural effects of psycholinguistic variables differed between visual and auditory presentation of the task stimuli, we included modality as an interaction term in our models (Figs. 1*D*, 3, 4).

### Variables of interest

Following a previous investigation (Eisenhauer et al., 2024), we focused on three types of variables of interest, analogous for auditory and visual modalities, representing three levels of linguistic processing: word, context, and sentence level. The word-level variables of interest were “word frequency” (i.e., lexical familiarity of each word), “word length” (number of letters or phonemes), and “phonological/orthographic distance” (i.e., phonological/orthographic familiarity). Context-level variables were “semantic similarity” of each word to the five preceding words in the sentence and “position” of each word in the sentence (i.e., amount of context available). Finally, “sentence processing” (the contrast between sentences and word lists, representing meaning, syntax, and combinatorial processes) was included as a sentence-level contrast of interest. Word frequency was obtained from the Subtlex-NL database (Keuleers et al., 2010), which contains Dutch word frequencies extracted from 44 million words from film and television subtitles, and log-transformed per million. Word length represented the number of letters in a written word or the number of phonemes of a spoken word, which was obtained by translating orthographic words into a phonemic transcript using the tool G2P (“grapheme-to-phoneme”; Reichel, 2012). Orthographic Levenshtein Distance 20 (Yarkoni et al., 2008) was computed using the R package vwr (Keuleers, 2013), and phonological distance was adapted from it by computing the average Levenshtein distance of the phonemic transcription of each word to the rest of words in the lexicon. Semantic similarity was calculated by estimating the cosine similarity between the vector representations of a word and the five words preceding it (De Boom et al., 2016). Vector representations were obtained from the “ELMo for many languages” model trained on Dutch (Fares et al., 2017; Che et al., 2018), based on the ELMo (“Embeddings from Language Models”) model (Peters et al., 2018), a computational language model trained on large text corpora, which represents words and phrases as vectors depending on their typical context—words which tend to appear in similar contexts will have more similar vector representations. Since semantic similarity of a word was calculated based on the five words that preceded it, it was not possible to obtain this measure for the first five words of the sentences, and thus we excluded them from our analyses. We also excluded words that did not have a meaning, such as function words or names, by focusing on content words. Words presented in word lists were also excluded, but word list processing was used for our sentence-level contrast of interest (see below, Effects of psycholinguistic variables on brain activation). All psycholinguistic measures were continuous variables except for this contrast of sentences versus word lists.

### Effects of psycholinguistic variables on brain activation

We followed the procedure used in the previous study that analyzed the visual version of the task (Eisenhauer et al., 2024), accessible in Open Science Framework (https://osf.io/5fxbd/). GLMs were used to estimate the effects of each of the psycholinguistic variables on brain activation, resulting in cortical maps showing the strength and direction of this effect. We estimated a GLM for each participant using FSL (version 6.0), including our “continuous context- and word-level parameters of interest” (semantic similarity, position, word frequency, word length, and phonological/orthographic distance) as explanatory variables (EVs). Following the methodology of previous studies that used the same dataset, our parameters of interest were modeled from the onset of each word with a fixed duration of 1 s, since the presentation duration of words was relatively short for an fMRI study and linguistic processing is likely to extend beyond the presentation duration (King et al., 2020; Eisenhauer et al., 2024). Part of speech was included as additional EVs, i.e., adjective, noun, verb, or others, with the last category comprising all remaining types of words such as names and function words. The following sentence-level EVs were also accounted for in the model, as variables of no interest: sentence with a complex relative clause, sentence without a complex relative clause, word list, and presentation order (position that a sentence occupied in reference to the others). The contrast between sentences and word lists was used as a “sentence-level parameter of interest” in the following analyses. Since we did not include context- and word-level EVs of word lists in our models due to high variance inflation factors (>10), we carried out separate models without context- and word-level EVs of words in sentences in order to control for their possible confounding effect in the contrast between sentences and word lists—these models showed similar results, confirming these EVs had no relevant effects in the contrast. Additionally, EVs comprising the questions and the fixation time window and cues that indicated the nature of the task block (sentence or word list) were included. The implicit baseline of the model consisted of two second blank periods between the sentences/word lists. All parametric EVs were centered and scaled, and EV time courses were convolved with a hemodynamic response function, including their temporal derivatives in the model. Finally, we also included 24 standard and extended motion parameters in the model, as well as voxels that were motion outliers based on framewise displacement. Variance inflation factors were below 10 for all EVs of interest and their derivatives.

By estimating these models at the first-level and carrying out a second-level average across participants for each EV using FSL, we obtained maps with two-tailed *z* values at the voxel level for the mean effects of our variables of interest. These maps were transformed from volumetric to surface space using the vol_to_surf function in nilearn. Vertices at the borders of the field of view were excluded if their *z* values were reduced by >25% due to surface-induced noise. *Z* values were averaged for the 400 surface parcels of a functional connectivity-based parcellation (Schaefer et al., 2018). Only parcels with available data for at least 25% of their surface vertices were included in the analysis. This and the limited field of view of fMRI task data led to missing *z* values in superior parieto-occipital and inferior anterior temporal regions, resulting in 270 parcels for the auditory task and 269 for the visual with available data for further analyses.

### Similarities between modalities in psycholinguistic variables’ brain effects

In order to investigate similarities between the effects of psycholinguistic variables for visual and auditory inputs, we carried out Pearson's correlations between the group-level maps of activation (mean *z* values across participants in 400 surface parcels) for the two modalities, for each psycholinguistic variable (e.g., correlation between the group-level map of activation for “auditory” sentence processing and the group-level map of “visual” sentence processing). Following a previous study (Eisenhauer et al., 2024), we assessed statistical significance—controlling for partial autocorrelation of the maps and type one error rate—by using a spin permutation procedure that generates null models by applying random rotations to spherical representations of surfaces (Alexander-Bloch et al., 2018). We obtained 1,000 permuted versions of the visual maps using BrainSpace (Vos de Wael et al., 2020). Correlations were considered significant only if their *t* value exceeded the 95th percentile of the *t* value distribution generated from the permuted maps, corrected for multiple comparisons with false discovery rating (FDR) using the Benjamini–Hochberg method.

### Modality differences in the relationship between variables and connectivity dimensions

We investigated if each psycholinguistic variable’s cortical map was related to the brain organization captured by the two first dimensions of whole-brain connectivity identified in a previous investigation (Margulies et al., 2016, Fig. 1*C*). These dimensions were obtained by transforming high-dimensional matrices of connectivity between brain regions to low-dimensional components representing the similarity or dissimilarity in connectivity patterns between regions. Specifically, Margulies et al. (2016) computed group-averaged matrices of connectivity between 32,492 nodes per hemisphere, based on time-series correlation during 15 min resting-state fMRI scans. They then calculated similarity between all pairs of rows using cosine distance, resulting in a matrix of weights between zero and one representing similarity of connectivity profiles among node pairs. Next, they used diffusion embedding to decompose this matrix to identify the components that explain its variance. The resulting components (or dimensions of connectivity, as we refer to them in this manuscript) are continuous variables that can be represented spatially in the brain, where the proximity of colors can be interpreted as greater similarity of connectivity patterns. Each dimension explains progressively less variance within the connectivity data, with the first two dimensions explaining 25 and 12%. Interestingly, the first dimension captures the separation (i.e., dissimilarity in connectivity patterns) between unimodal sensory–motor systems and heteromodal regions (Figs. 1*C*, 3), while the second dimension separates the auditory and somatomotor cortices from visual regions (Figs. 1*C*, 4). For our analyses, we used these two first dimensions, using the maps from BrainSpace (Vos de Wael et al., 2020).

For each variable of interest and each modality separately, we used linear models in R (version 4.0.4; https://www.r-project.org/) to compute the association between psycholinguistic effects (maps' *z* values) with the two first dimensions of connectivity, across the brain parcels with available psycholinguistic activity data. To explore if nonlinear models better explained the relationship between the dimensions and each variable's effect, we compared a model that included the quadratic term of the variable in addition to the initial linear effect by using nested model comparisons, based on an *F* test using the ANOVA function in R (Eisenhauer et al., 2024). If the comparison showed a significant difference and the effect of the quadratic term was statistically significant, this indicated that the more complex quadratic model better explained the relationship; otherwise, the linear model should be favored.

To investigate modality differences, we collapsed visual and auditory data and included modality as an interaction term in our models. We assessed the statistical significance of the main effect of the dimension and its interaction with modality, controlling for spatial autocorrelation and Type 1 error rate with a spin permutation procedure (Alexander-Bloch et al., 2018), using 1,000 permuted versions of the dimensions obtained using BrainSpace (Vos de Wael et al., 2020). A main effect of dimension or an interaction with modality was considered significant if its *t* value exceeded the 95th percentile of the *t* value distribution generated from the permuted dimensions and was FDR corrected for multiple comparisons.

## Results

### Similarity between modalities in the effects of psycholinguistic variables on brain activation

We found positive correlations between macroscale brain responses relating to sentence and semantic processing for visual and auditory inputs (Fig. 2). The contrast of sentences versus word lists (*r* = 0.81; *p*_spin_ < 0.006; FDR corrected) and the effect of semantic similarity (*r* = 0.33; *p*_spin _= 0.006; FDR corrected) both showed this similarity across modalities, suggesting that sentence processing effects are heteromodal with common cortical spatial patterns for written and spoken sentences. We carried out complementary exploratory correlations “within” each modality, and, interestingly, these effects were not similar to each other: “visual” sentence and semantic processing for written words did not show a spatial correlation at the macroscale, and nor did “auditory” sentence and semantic processing variables (visual, *r* = −0.13; *p*_spin_ > 0.05; auditory, *r* = −0.21; *p*_spin_ > 0.05; FDR corrected). Figure 2 shows that ATL responded more to sentences compared with word lists, yet it also responded more when the words in the sentences were less similar in meaning.

In contrast, word position, frequency, and length effects showed no significant similarity across visual and auditory inputs (*r* =0.06; *p*_spin_ > 0.05; *r* = 0.08; *p*_spin_ > 0.05; *r* = −0.17; *p*_spin_ > 0.05; Fig. 2), suggesting these lexical and context effects do not reflect heteromodal processes that are shared across modalities.

Phonological and orthographic distance values for words presented in spoken and written sentences were positively correlated (*r* = 0.94), but their effects on brain activity were negatively correlated, indicating that brain regions that responded more when written words were orthographically similar to other words also responded less when spoken words were phonologically similar to other words (*r* = −0.24; *p*_spin_ = 0.04; FDR corrected; Fig. 2). This acoustic/visual variable had opposing effects across modalities in both primary and heteromodal regions—lateral visual cortex responded more strongly to less familiar orthographic forms (when orthographic distance was greater), while heteromodal DMN regions responded more strongly to more familiar letter patterns (when orthographic distance was lower), suggesting that recognition of words with more unique forms elicited more input-driven demands. In contrast, regions proximal to the auditory and motor cortex showed a weaker response when phonological forms were less similar to other words (when phonological distance was greater), and DMN regions showed a stronger response, presumably because the recognition of transient and confusable auditory words was easier when the inputs were more unique and less overlapping with other words.

### Intramodality relationship with the first connectivity dimension capturing the heteromodal–unimodal distinction

The first dimension of whole-brain intrinsic connectivity captures the functional separation between heteromodal and unimodal cortices, with intermediate dimensional values located in regions related to cognitive control and attention, i.e., salience, dorsal, and ventral attention networks (Margulies et al., 2016; Yeo et al., 2011; Fig. 1*C*). We explored both linear and quadratic relationships between this connectivity dimension and the brain activity associated with each psycholinguistic variable, which allowed us to identify situations in which the ends of the dimension showed opposing responses (i.e., positive effects at one end of the dimension vs negative effects at the other end, for linear effects), or more similar responses toward the unimodal and heteromodal ends of the dimension, in contrast to a different response in attention and control regions nearer the middle of the dimension (for quadratic effects). We report quadratic models when the comparison between the model that included the quadratic term and the exclusively linear model showed a significant difference and the main effect of the quadratic term was significant. When this was not fulfilled, linear models are reported.

The first dimension of whole-brain connectivity showed a significant relationship with the pattern of brain activation for the following “auditory” psycholinguistic variables: sentence processing (*t* = 3.90; *p*_spin_ < 0.005; FDR corrected), word position (*t* = 6.33; *p*_spin_ < 0.005; FDR corrected), word length (*t* = −3.95; *p*_spin_ < 0.005; FDR corrected), and phonological distance (*t* = 4.77; *p*_spin_ < 0.005; FDR corrected; Fig. 3). This means that these variables elicited a pattern of activation that was spatially aligned with the unimodal–heteromodal dimension. Phonological distance and position showed a linear relationship with this dimension, suggesting an asymmetry in the involvement of input-driven and heteromodal processing for these auditory variables: words that were more phonologically distant showed higher activation toward the heteromodal end, and words that appeared in an earlier position in the sentence showed more activation toward the unimodal end, probably reflecting easier recognition dependent on high uniqueness and context availability for auditory stimuli. A quadratic relationship was found between the first dimension and sentence processing (*F* = 10.62; *p*  <  0.001; FDR corrected) and word length (*F* = 12.31; *p* < 0.001; FDR corrected), with symmetric involvement of both ends for shorter words and sentences; longer words and word lists resulted in higher activation in intermediate regions of the dimension, probably reflecting more cognitive control and attentional demands for this type of stimuli.

The first connectivity dimension also showed a significant relationship with the following “visual” variables' brain effects: sentence processing (*t* = 4.35; *p*_spin_ < 0.005; FDR corrected), word frequency (*t* = 5.42; *p*_spin_ < 0.005; FDR corrected), word length (*t* = 6.76; *p*_spin_ < 0.005; FDR corrected) and orthographic distance (*t* = −6.59; *p*_spin_ < 0.005; FDR corrected). Orthographic distance showed a linear relationship with this dimension: brain activation was higher toward the unimodal end of the first dimension for more orthographically distant words. This is the opposite pattern to the phonological distance variable, perhaps because recognition of more common letter combinations requires less sensory processing. Word length also showed the opposite pattern to its analogous auditory variable, although still quadratic (*F* = 36.18; *p* < 0.001; FDR corrected): shorter words produced higher activation in intermediate regions of the first dimension, associated with networks that underpin attention and cognitive control, while longer words induced stronger activation toward both sensory and heteromodal ends of this dimension. Finally, a quadratic relationship was found between the first dimension and word frequency (*F* = 22.76; *p* < 0.001; FDR corrected) and sentence processing (*F* = 18.91; *p* < 0.001; FDR corrected), pointing to a symmetrical involvement of both heteromodal and sensory–motor ends of this dimension for more frequent words and connected sentences, with less frequent words and word lists eliciting more activation in intermediate regions associated with attention and cognitive control. No significant effects were found for the rest of psycholinguistic variables.

### Intramodality relationship with the second connectivity dimension capturing the distinction between visual and auditory–motor cortices

The second dimension of whole-brain connectivity reflects the separation of auditory and somatomotor from visual primary cortices (Fig. 1*C*). Again, we explored both linear and quadratic relationships between psycholinguistic variables’ brain activity and this connectivity dimension, to identify circumstances in which the visual and auditory–motor ends of the dimension showed symmetric or asymmetric involvement in language processing.

The second connectivity dimension was significantly related to sentence processing (*t* = 3.73; *p*_spin_ < 0.005; FDR corrected), semantic similarity (*t* = −6.15; *p*_spin_ = 0.009; FDR corrected), position (*t* = −4.80; *p*_spin_ = 0.024; FDR corrected), and word length (*t* = 5.65; *p*_spin_ = 0.017; FDR corrected) only in the “auditory” modality (Fig. 4). Nonsignificant trends were found for word frequency (*t* = −3.99; *p*_spin_ = 0.078; FDR corrected) and phonological distance (*t* = −3.15; *p*_spin_ = 0.051; FDR corrected) only in the auditory modality. Longer and less semantically similar words produced higher activation toward the auditory end of the dimension following a linear pattern, suggesting an asymmetry in the recruitment of auditory and visual primary regions for words that are potentially more difficult to recognize. However, sentence processing and words with an earlier position in the sentence showed higher activation in both auditory and visual ends of the second dimension, relative to the middle, following a quadratic pattern (sentence processing, *F* = 18.41; *p* < 0.001; FDR corrected; position, *F* = 15.54; *p* < 0.001; FDR corrected), which suggests similar recruitment of the two primary systems for these aspects of processing. No significant relationships were found between the second dimension and any effects of psycholinguistic variables on brain activation in the visual modality.

### Modality differences in the relationship between psycholinguistic variables and connectivity dimensions

In order to investigate modality differences, we constructed models including both auditory and visual data and included modality as an interaction term. A significant interaction indicated a different relationship between the connectivity dimension and the psycholinguistic variable’s effect on brain activation for spoken and written words.

A significant interaction between the first dimension of whole-brain connectivity and modality on brain activation was found for the following psycholinguistic variables: word length (*t* = 11.30; *p*_spin_ = 0.006; FDR corrected) and phonological/orthographic distance (*t* = −11.44; *p*_spin_ = 0.006; FDR corrected; Fig. 3). We found opposing effects for auditory and visual inputs: listening to shorter and more phonologically distant spoken words resulted in more activation toward the heteromodal end of the dimension, but the heteromodal cortex also showed higher activation when reading longer and less orthographically distant words, demonstrating that word length and phonological/orthographic distance have different effects on the neural states that underpin language processing, depending on the modality of presentation for words in a sentence.

The second dimension of whole-brain connectivity interacted with modality for the following variables: semantic similarity (*t* = 6.84; *p*_spin_ = 0.006; FDR corrected) and phonological/orthographic distance (*t* = 5.83; *p*_spin_ = 0.017; FDR corrected; Fig. 4). Less semantically similar words elicited more activation toward the auditory end of the second dimension for auditory compared with written sentences, pointing toward a more unbalanced recruitment of primary sensory systems for spoken than written words when semantic predictions are not fulfilled. Less phonologically distant words also showed more activation toward both visual and auditory–motor ends of the dimension (this pattern was trending; see Intramodality relationship with the second connectivity dimension capturing the distinction between visual and auditory–motor cortices), while there were no effects for orthographic distance, suggesting that more common word patterns that are more confusable place higher demands on all primary sensory regions when language inputs are presented in the auditory modality as opposed to the visual modality. No statistically significant differences were found for the other psycholinguistic variables.

## Discussion

Language comprehension involves distinct processes thought to be hierarchically ordered and that have been localized to specific brain areas (Friederici, 2011; Price, 2012; Carreiras et al., 2014). However, it is increasingly recognized that brain regions support multiple facets of cognition and that cognition draws on the whole brain (Mesulam, 1990; Anderson, 2010; Margulies et al., 2016). We investigated macroscale activity induced by psycholinguistic variables linked to language input processes and meaning for visually and auditorily presented sentences. We then decomposed the relationships between these effects and the principal dimensions of intrinsic connectivity. This dimensional approach provides complementary information to traditional methods used to study the neurobiology of language, since it describes language processing holistically (avoiding the need for artificial regions of interest) and identifies “systematic” functional transitions between regions (which are repeated across disparate regions, e.g., transitions between unimodal and heteromodal cortices are seen in both lateral temporal and medial prefrontal cortices). We found that macroscale patterns of brain activity were similar across modalities for sentence-level and semantic variables, in line with previous evidence that higher-order language processing involves common processes recruited by both auditory and visual stimuli (Uddén et al., 2022). However, effects of orthographic and phonological distance on brain activation were negatively correlated between modalities, and the first connectivity dimension showed opposite effects for word length and orthographic/phonological distance for spoken and written words. Moreover, the second connectivity dimension was only correlated with brain activation in the auditory modality, and there was an asymmetry in the recruitment of primary processing systems when listening to longer and more semantically dissimilar words. These findings further support the idea that fundamental differences between the nature of auditory and visual linguistic stimuli influence language processing (Cohen et al., 2009; Robinson et al., 2018).

A key finding was the positive correlation between modalities in macroscale activation patterns linked to sentence processing and semantic similarity. This aligns with previous research on the same dataset that demonstrated heteromodal syntactic responses at the regional level (Uddén et al., 2022). The ATL showed strong yet distinct responses to sentence processing and semantic similarity: it was more active for sentences than for word lists, consistent with earlier results comparing sentences to a low-level baseline in this dataset (Uddén et al., 2022), and it showed increased activation when semantic similarity was low with the rest of the sentence, reflecting greater semantic retrieval demands. These results also converge with prior studies implicating the ATL in sentence processing (Price, 2012) and heteromodal semantic representation (Jackson et al., 2016; Lambon-Ralph et al., 2017). Together, these results suggest that the ATL is sensitive both to the variability of individual word meanings and to the overall coherence of linguistic input.

Although phonological and orthographic distances were positively correlated, the macroscale patterns of brain activation induced by these analogous psycholinguistic variables showed a negative correlation, revealing opposite spatial activation patterns. Similarly, the first dimension of whole-brain connectivity—separating heteromodal and unimodal cortices—showed opposing effects of orthographic and phonological distance: although both variables were modulated in a linear manner, the effect was positive for phonological distance and negative for orthographic distance. In other words, recognition of spoken words is dependent on the unimodal end of the dimension when words have a similar auditory form, whereas for written words, it is unique visual forms that recruit the unimodal end. The modulation of the unimodal–heteromodal dimension by word length also showed opposite patterns for spoken and written modalities, although the effects were quadratic: longer spoken words relied more on attentional and control regions, while longer written words recruited both sensory and memory-based heteromodal processes associated with the DMN. This suggests that word length in the context of a sentence poses more encoding demands only in the auditory modality, while it elicits greater support from input information and lexical–semantic knowledge in the visual modality.

These opposing effects can be explained in terms of the “noisy channel model of communication,” which emphasizes that noise is present in typical language use and sentence comprehension cannot be explained without considering this imperfect input (Gibson et al., 2013; Dautriche et al., 2017). Linguistic auditory stimuli (i.e., spoken words) are highly susceptible to misperception in many conditions—they are transient in nature, only briefly available for processing, and typically present in noisy environments, with other stimuli in the same channel competing for processing resources. Therefore, spoken words that have a common phonological form can be difficult to differentiate from others (Suárez et al., 2011)—many similar-sounding words are simultaneously activated leading to competition in lexical access (Scarborough and Zellou, 2013). This may elicit high input-processing demands, compared with words that have a more unique sound and are more easily recognizable. Supporting evidence is provided by behavioral studies showing that large phonological neighborhoods are detrimental for spoken word recognition (Luce and Pisoni, 1998), while words with many neighbors are articulated more clearly, suggesting they require more clarity in communication (Scarborough and Zellou, 2013). In contrast, visual linguistic material tends to be present in less noisy and ambiguous conditions, and written words that have a more frequent form are more efficiently recognized (Yarkoni et al., 2008) because of the familiarity of their letter combinations, making them more dependent on heteromodal memory processes. In line with this proposal, a self-paced reading study using a subset of sentences from the dataset we used (Kapteijns and Hintz, 2021) found that orthographic distance was positively correlated with reading times; i.e., words with common orthographic forms were processed faster (Eisenhauer et al., 2024). Our findings for the second connectivity dimension also support the idea that auditory and visual language inputs differ in how prone they are to misperception and that this shapes large-scale brain responses to word-level features. When participants listened to long or semantically dissimilar words, there was stronger activation at the auditory–motor end of the second dimension and weaker engagement of the visual end. This pattern—a linear shift between sensory systems—emerged only for the auditory modality, highlighting an asymmetry in how primary processing regions are recruited when listening to sentences.

The first dimension of intrinsic whole-brain connectivity also related to macroscale patterns of activation that were largely the same for visual and auditory inputs. We found a balanced contribution of unimodal and heteromodal ends of this dimension (i.e., a quadratic effect) for sentence processing in both modalities: word lists activated more intermediate regions of the dimension, perhaps because the higher attentional and control demands of this material activate attentional and control systems toward the middle of this dimension (Margulies et al., 2016), while sentences resulted in high activation at both heteromodal and unimodal ends, in line with previous results focusing on regional activation when processing syntax and meaning at the sentence level (Humphries et al., 2006; Matchin et al., 2017). This lack of difference between modalities supports the supramodal nature of sentence processing, as opposed to word-level characteristics, which were affected differently depending on modality. We also found a positive relationship between position of spoken words in the sentence and activation toward the heteromodal end, in line with previous evidence in the same dataset showing more activation in syntactic processing areas toward the end of the sentence (Uddén et al., 2022). This suggests that neural mechanisms underlying context-dependent meaning and syntax are also shared across modalities.

Although our study provides evidence for both linear and nonlinear relationships between dimensions of intrinsic connectivity and brain activation patterns, these relationships could follow more complex patterns than the ones we explored. However, our models examining simple spatial patterns capture the most important trends in the data. The absence of information in several parcels of the brain due to the reduced field of view in the original study also limits the extent of our interpretations, particularly in the dorsal parietal cortex; nevertheless, the focus of this study was to investigate how macroscale brain states depend on whole-brain connectivity dimensions, in contrast to adopting a regional approach. Future research could examine the extent to which regional analyses differ from the whole-brain perspective, while time-resolved methodologies could reveal patterns of activation across connectivity dimensions that evolve over time (Eisenhauer et al., 2024). Finally, it would be valuable to examine whether individual differences in brain responses to psycholinguistic variables—or in how closely these responses align with connectivity dimensions—predict variation in language processing ability; we could not test this due to the absence of suitable behavioral data.

Perhaps the most important contribution of this study is to help bridge the gap between traditional cognitive neuroscience approaches to language and newer dimensional models of brain organization. The gradient approach offers complementary insights by characterizing systematic transitions in functional connectivity across the cortex, such as shifts from unimodal to heteromodal regions, without requiring artificial divisions into discrete regions of interest. These macroscale patterns are not exclusive to language, but they have important implications for this domain given that it extends from sensory–motor processing to abstract semantics (Price, 2012). The relationships we found between connectivity dimensions and psycholinguistic effects on brain activation are consistent with the view that complex functions, including language, emerge from activity of the whole brain (Skipper et al., 2022; Shao et al., 2022; Aliko et al., 2023). Moreover, connectivity dimensions allow us to describe language processing holistically using a small number of parameters to describe key topographical patterns across the cortex. Crucially, this dimensional framework helps to explain how individual brain areas participate in multiple functions, depending on their relative position along different connectivity axes; for example, differences between sensory modalities may emerge on one gradient, while conceptual abstraction varies along another. By leveraging this dimensional perspective, we can provide a more holistic understanding of the functional architecture that supports language processing in the brain.

In summary, our study shows that the dimensions underlying whole-brain intrinsic connectivity modulate macroscale patterns of activity evoked by psycholinguistic variables in a different fashion depending on the level of processing (sentence, context, or word) and modality (visual or auditory). Specifically, patterns of macroscale brain activity elicited by word-level variables differ in their relationship to the first dimension of connectivity depending on modality, as opposed to sentence- and context-level variables, showing more consistency across modalities. Furthermore, the second dimension reveals an imbalance between primary systems that is only present when processing auditory stimuli. In this way, our findings delineate both balance and asymmetry in functional brain systems during language comprehension and highlight the relevance of investigating brain patterns at the macroscale level.
